# Mechanisms of Aerobic Exercise Upregulating the Expression of Hippocampal Synaptic Plasticity-Associated Proteins in Diabetic Rats

**DOI:** 10.1155/2019/7920540

**Published:** 2019-02-18

**Authors:** Jingjing Li, Yuran Liu, Beibei Liu, Feng Li, Jingyun Hu, Qian Wang, Mingming Li, Shujie Lou

**Affiliations:** ^1^Shanghai University of Sport, Shanghai, China; ^2^Brain and Spinal Cord Innovative Research Center, Tongji Hospital, Department of Physiology and Pharmacology, Tongji University School of Medicine, Shanghai, China; ^3^Clinical Medicine Department, Weifang Medical College, Weifang, China; ^4^Guangxi Normal University for Nationalities, Chongzuo, China

## Abstract

We investigated the effects of aerobic exercise on the expression of hippocampal synaptic plasticity-associated proteins in rats with type 2 diabetes and their possible mechanisms. A type 2 diabetes rat model was established with 8 weeks of high-fat diet combined with a single intraperitoneal injection of streptozotocin (STZ). Then, a 4-week aerobic exercise intervention was conducted. Memory performance was measured with Y maze tests. The expression and activity of synaptic plasticity-associated proteins and of proteins involved in the PI3K/Akt/mTOR, AMPK/Sirt1, and NF*κ*B/NLRP3/IL-1*β* signaling pathways were evaluated by western blot. Our results show that aerobic exercise promotes the expression of synaptic plasticity-associated proteins in the hippocampus of diabetic rats. Aerobic exercise also activates the PI3K/Akt/mTOR and AMPK/Sirt1 signaling pathways and inhibits the NF*κ*B/NLRP3/IL-1*β* signaling pathway in the hippocampus of diabetic rats. Therefore, modulating the PI3K/Akt/mTOR, AMPK/Sirt1, and NF*κ*B/NLRP3/IL-1*β* signaling pathways is probably the mechanism of aerobic exercise upregulating the expression of hippocampal synaptic plasticity-associated proteins in diabetic rats.

## 1. Introduction

Our previous metabolomics study found impaired glucose metabolism in the hippocampus of diabetic rats, mainly indicated by inhibition of the TCA (tricarboxylic acid) cycle and activation of the glycolysis pathway, polyol pathway, and pentose phosphate pathway [1]. This suggests that glucose metabolism in the hippocampus of diabetic rats tends to shift from the aerobic oxidative metabolic pathway to other pathways. However, the consequences of this change and its connection to diabetes-related cognitive dysfunction remain unknown.

In diabetic animals, peripheral blood glucose concentration rises and peripheral insulin resistance occurs. It is noteworthy that, at the same time, glucose concentration in the hippocampus also rises. This high-glucose environment may inhibit mitochondrial function, leading to massive production of reactive oxygen species [2]. Moreover, mitochondrial dysfunction and overproduction of reactive oxygen species are important mechanisms for the development of hippocampal insulin resistance [3]. More and more studies suggested that the PI3K (phosphatidylinositol 3-kinase)/Akt (protein kinase B, PKB) signaling pathway is an important factor of synaptic plasticity. As an important downstream signaling molecule of PI3K/Akt, mTOR (mechanistic target of rapamycin) is also involved in the regulation of the expression of synaptic plasticity-associated proteins [4]. As SYN (synaptophysin), Homer, and NMDAR (N-methyl-D-aspartic acid receptor) are all important presynaptic (SYN) or postsynaptic (Homer and NMDAR) proteins involved in synaptic plasticity and closely related to cognition, it has been reported that hippocampal SYN and NMDAR1 expressions were downregulated in diabetic rats or AD (Alzheimer's disease) mice [5–8]. However, it remains unclear whether the alterations of hippocampal glucose metabolism in type 2 diabetes affect the insulin signaling pathway and how this would affect the expression of synaptic plasticity-associated proteins.

The brain requires high energy for proper function. Normally, the glucose TCA cycle is the predominant pathway for energy production. Although the glycolytic pathway can also generate ATP (adenosine triphosphate), its efficiency is much lower. Under diabetic conditions, the hippocampal TCA cycle declines, reducing ATP generated through this pathway. Although the hippocampal glycolytic pathway was enhanced, whether ATP generated through this pathway can satisfy the metabolic needs of the brain cannot be determined. Since brain energy mainly supplies for synaptic transmission, adequate energy supply in the brain is crucial for the maintenance of synaptic plasticity [9]. As important energy-sensitive proteins, AMPK (adenosine 5′-monophosphate-activated protein kinase) and Sirt1 were reported to play an important role in maintaining synaptic plasticity [10–12]. In addition, AMPK and Sirt1 (silent mating type information regulation 2 homolog-1) can also participate in the regulation of inflammation [13, 14]. Yet, whether abnormal hippocampal glucose metabolism leads to the imbalance of energy metabolism and inflammation in diabetic rats and whether such effects are related to a decreased expression of synaptic plasticity-associated proteins need further investigation.

Aerobic exercise is considered a good strategy to alleviate diabetes symptoms and to improve cognitive function. Studies have shown that aerobic exercise can promote energy metabolism, reduce insulin resistance, and participate in the glucose homeostasis regulation [15–17]. In addition, aerobic exercise can promote the expression of synaptic plasticity-associated proteins by reducing the inflammatory cytokines such as IL-1*β* (interleukin-1*β*) and TNF-*α* (tumor necrosis factor-*α*) in the hippocampus [18, 19]. However, under diabetic conditions, the regulatory effects of aerobic exercise and its mechanism are not yet fully understood. We hypothesized that these effects are related to improved glucose metabolism in the hippocampus of diabetic rats induced by aerobic exercise. Possible mechanisms of aerobic exercise upregulating the expression of synaptic plasticity-associated proteins include activation of the insulin signaling pathway, improvement in energy metabolism, and inhibition of inflammation.

Based on the above research issues, we established a rat model of type 2 diabetes through a high-fat diet in combination with a single intraperitoneal injection of STZ. We studied the insulin signaling pathway PI3K/Akt/mTOR, energy metabolism-related AMPK/Sirt1 signaling pathway, and inflammatory signaling pathway NF*κ*B/NLRP3 (NLR family, pyrin domain containing 3)/IL-1*β* in the hippocampus, aiming to reveal the effects of diabetes on the expression of synaptic plasticity-associated proteins and their possible mechanisms.

## 2. Materials and Methods

### 2.1. Establishment of a Type 2 Diabetes Model and Aerobic Exercise Training

A total of 102 male Sprague-Dawley (SD) rats with weight ranging from 190 to 210 g were purchased from Beijing Weitong Lihua Experimental Animal Technical Co. Ltd. (Certificate of Conformity: SCXK (Beijing) 2012-0001). We followed the methods of Li et al. [1] to establish a type 2 diabetes model (the animals were randomly divided into two groups with similar body weight: normal chow diet group (C, *n* = 8) and high-fat diet group (HF, *n* = 94)). At the end of the 8th week, the mean body weight of the HF group was 20% heavier than that of the C group, indicating that the obese model was established. The obese rats were intraperitoneally injected with STZ (30 mg/kg body weight in a 0.1 mmol/l citrate buffer, pH 4.5) while the control rats with 0.1 mmol/l citrate buffer at the same dose. At the 3rd and 7th day of STZ injection, the rats with fasting blood glucose concentration (Roche glucometer, Germany) higher than 11.1 mmol/l were considered diabetic and carried out 4 weeks (6 days/week) of aerobic exercise (TDM rats were adapted to the treadmill for 3 days (the running speed was 15 m/min for 15 min)) and then followed by 4 weeks (6 days/week) of aerobic exercise. In the 1st week, the rats run at 15 m/min for 30 min per day; in the 2nd week, the rats run at 15 m/min for 60 min per day; in the 3rd week, the rats run at 20 m/min for 60 min per day; and in the 4th week, the rats run at 20 m/min for 90 min per day.

### 2.2. Behavioral Test

**Figure 1:**
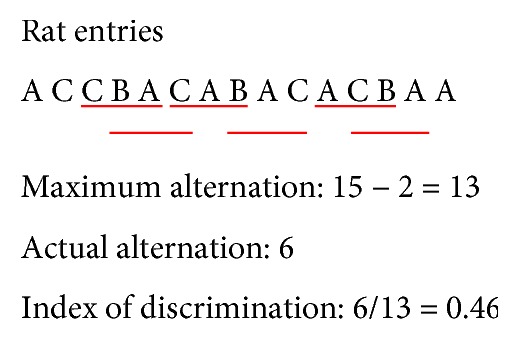
Sample spontaneous alternation test. Red lines mark the actual alternations in the 15-entry sample test result.

We assessed the rats' memory performance with the Y maze. In the spontaneous alternation test, a rat was introduced to the center of the maze. The rat was allowed to freely explore the three arms (A, B, and C) for 5 min. The number of arm entries and the number of triads were recorded to calculate the percentage of alternation. Spontaneous alternation (i.e., index of discrimination) is defined as the ratio of successive entries into each of the three arms on triplet sets with overlaps (actual alternations) to maximum alternations (total arm entries—2) × 100 (Figure 1). We also performed a novel object recognition test with the following procedures. The rat starts at the end of one arm (start arm), then chooses one of the other two arms. One arm of the Y maze is blocked off, and the rat is allowed to explore the other two arms for 5 min. The rat was returned to the maze 2 h later with all arms open and kept in the maze for 5 min. The number of entries into each arm was recorded. More entries into the novel arm signify better memory ability.

### 2.3. Sample Collection, Examination, and Western Blot

After 4 weeks of aerobic exercise intervention, the rats were sacrificed after anesthesia with chloral hydrate; their blood samples were collected. The fasting serum glucose and insulin were measured by a Roche glucometer and Rat Ins1/Insulin ELISA Kit (no. RAB0904, Sigma-Aldrich, USA), respectively. The bilateral hippocampi were quickly extracted from the brain and frozen in liquid nitrogen. The right hippocampi were used for western blot (6 rats were randomly selected from each group).

Hippocampal proteins were extracted with an ice-cold Membrane Nuclear and Cytoplasmic Protein Extraction kit (no. C510002, Sangon Biotech, Shanghai, China), and their concentrations were determined with a BCA kit (BCA Protein Assay Kit, P0010, Beyotime, Shanghai, China). The details were demonstrated in our previous study [14]. For the protein assay, protein samples containing an equal amount of protein (30 *μ*g) were electrophoresed on SDS-PAGE gels and transferred to PVDF membranes. The membranes were blocked with 5% BSA in a TBST buffer and incubated overnight at 4°C with different primary antibodies: anti-Homer 1 (1 : 1000, Cell Signaling Technology, #8231, USA), anti-NMDAR1 (1 : 1000, Abcam Corporation, ab109182, UK), anti-synaptophysin (1 : 500, Abcam Corporation, ab8049, UK), p-PI3K (1 : 1000, Cell Signaling Technology, #4228, USA), PI3K (1 : 1000, Cell Signaling Technology, #4292, USA), p-Akt (1 : 1000, Cell Signaling Technology, #4060, USA), Akt (1 : 1000, Cell Signaling Technology, #9272, USA), p-AMPK*α* (1 : 1000, Cell Signaling Technology, #2535, USA), AMPK*α* (1 : 1000, Cell Signaling Technology, #5831, USA), p-mTOR (1 : 1000, Cell Signaling Technology, #2971, USA), mTOR (1 : 1000, Cell Signaling Technology, #2972, USA), raptor (1 : 1000, Cell Signaling Technology, #2280, USA), rictor (1 : 1000, Cell Signaling Technology, #2114, USA), p-p70s6k (1 : 1000, Cell Signaling Technology, #9234, USA), p70s6k (1 : 1000, Cell Signaling Technology, #2708, USA), p-4EBP2 (1 : 200, Santa Cruz Biotechnology, sc-23767-R, USA), 4EBP2 (1 : 1000, Cell Signaling Technology, #2845, USA), Sirt1 (1 : 1000, Cell Signaling Technology, #2496, USA), anti-NF*κ*B p65 (acetyl-K310) antibody (1 : 1000, Abcam Corporation, ab19870, UK), NF*κ*B (1 : 1000, Cell Signaling Technology, #8242, USA), IL-1*β* (1 : 1000, Abcam Corporation, ab9722, UK), and NLRP3 (1 : 1000, Novus, NBP2-12446, USA). *β*-Actin (1 : 1000, Proteintech, 20536-1-AP, USA) and *β*-tubulin (1 : 1000, Proteintech, 10068-1-AP, USA) were used as the loading control of the cytoplasm or membrane proteins, and Lamin B1 (1 : 1000, Proteintech, 12987-1-AP, USA) was used as the loading control of nuclear proteins. After rinsing with TBST, the membranes were incubated with a secondary antibody, peroxidase-conjugated goat anti-rabbit IgG (H+L) (1 : 5000, Proteintech, SA00001-2, USA) (which binds to the primary antibodies binding to target proteins and becomes luminous), for 1 h at room temperature. The membranes were developed with an advanced reagent (Millipore, USA), and the protein bands were visualized with an automatic chemiluminescence apparatus (Tanon Biotechnology, China). The densities of the bands were determined with the ImageJ software.

### 2.4. Statistical Analysis

Data were processed by SPSS 20.0 and represented as mean ± SD. Statistical significance was analyzed by one-way ANOVA; the differences with *p* < 0.05 were considered significant.

## 3. Results

### 3.1. Aerobic Exercise Reduces Fasting Blood Glucose and Improves Insulin Resistance

Compared with the C rats, DM rats exhibited significantly higher concentrations of FBG, FINS, and HOMA-IR (Table 1), suggesting that hyperglycemia and peripheral insulin resistance occurred in diabetes. After 4 weeks of aerobic exercise intervention, FBG, FINS, and HOMA-IR in the TDM rats were significantly lower than those in the DM rats, suggesting that aerobic exercise can reduce fasting blood glucose and peripheral insulin resistance.

### 3.2. Aerobic Exercise May Improve Memory of Diabetic Rats

In the spontaneous alternation test, the number of actual alternations and the number of maximum alternations of DM rats were significantly lower than those of C rats (Table 2). In the novel object recognition test, the total number of arm entries and the number of novel arm entries of DM rats were both markedly lower compared to those of C rats (Table 3). These results indicate that diabetic rats experience memory impairment. All the above indexes of TDM rats show a tendency to increase compared to those of DM rats, suggesting that aerobic exercise may improve the memory of diabetic rats.

### 3.3. Aerobic Exercise Promotes the Expression of Synaptic Plasticity-Associated Proteins in the Hippocampus of Diabetic Rats

**Figure 2:**
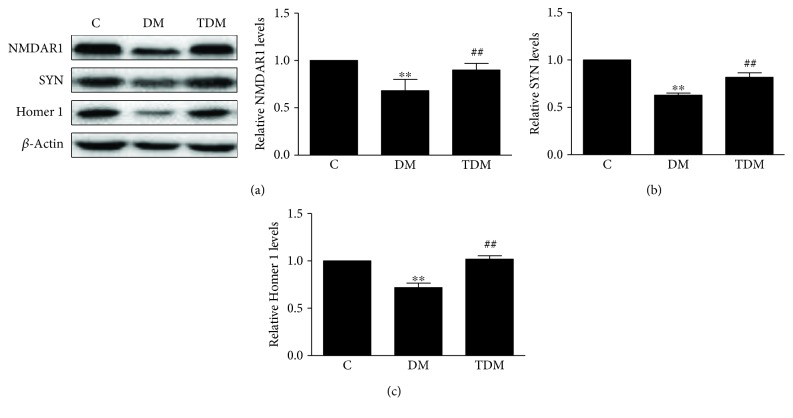
Hippocampal synaptic plasticity-associated proteins in diabetic rats and the effects of aerobic exercise. Levels of NMDAR1 (a), SYN (b), and Homer 1 (c) were significantly lower in diabetic rats. After aerobic exercise intervention, these protein levels were significantly increased. ^∗∗^*p* < 0.01, DM group vs. C group; ^##^*p* < 0.01, TDM group vs. DM group.

We measured the levels of NMDAR1, SYN, and Homer 1 in the hippocampus. These are important synaptic plasticity-associated proteins, beneficial for memory. The levels of NMDAR1, SYN, and Homer 1 in DM rats were significantly lower than those in C rats, and the levels of the above proteins in TDM rats were higher than those in DM rats (Figure 2). The data indicates that diabetes reduces the levels of synaptic plasticity-associated proteins in the hippocampus; this decrease can be reversed by aerobic exercise.

### 3.4. Aerobic Exercise Activates the PI3K/Akt/mTOR Signaling Pathway in the Hippocampus of Diabetic Rats

**Figure 3:**
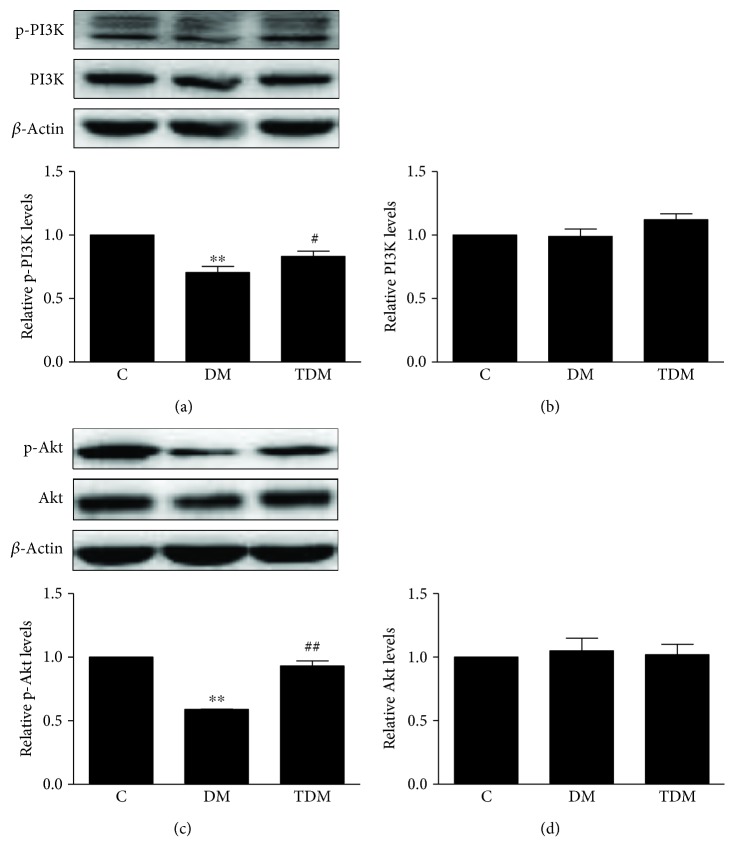
Hippocampal PI3K/Akt signaling pathway in diabetic rats and the effects of aerobic exercise. Type 2 diabetes significantly inhibits the activation of hippocampal PI3K (a) and Akt (c). Aerobic exercise can reverse these changes. ^∗∗^*p* < 0.01, DM group vs. C group; ^#^*p* < 0.05, ^##^*p* < 0.01, TDM group vs. DM group.

We measured the levels of p-PI3K and p-Akt in the hippocampus of diabetic rats and estimated the regulation of aerobic exercise. The results show that the levels of p-PI3K and p-Akt in DM rats were lower compared to those in C rats and the levels of p-PI3K and p-Akt in TDM rats were higher than those in DM rats (Figure 3). The results indicate that diabetes inhibits the activity of PI3K and Akt, while aerobic exercise activates the PI3K/Akt signaling pathway.

### 3.5. Aerobic Exercise Upregulates the AMPK/Sirt1 Signaling Pathway and Downregulates the NF*κ*B/NLRP3/IL-1*β* Signaling Pathway in the Hippocampus of Diabetic Rats

**Figure 5:**
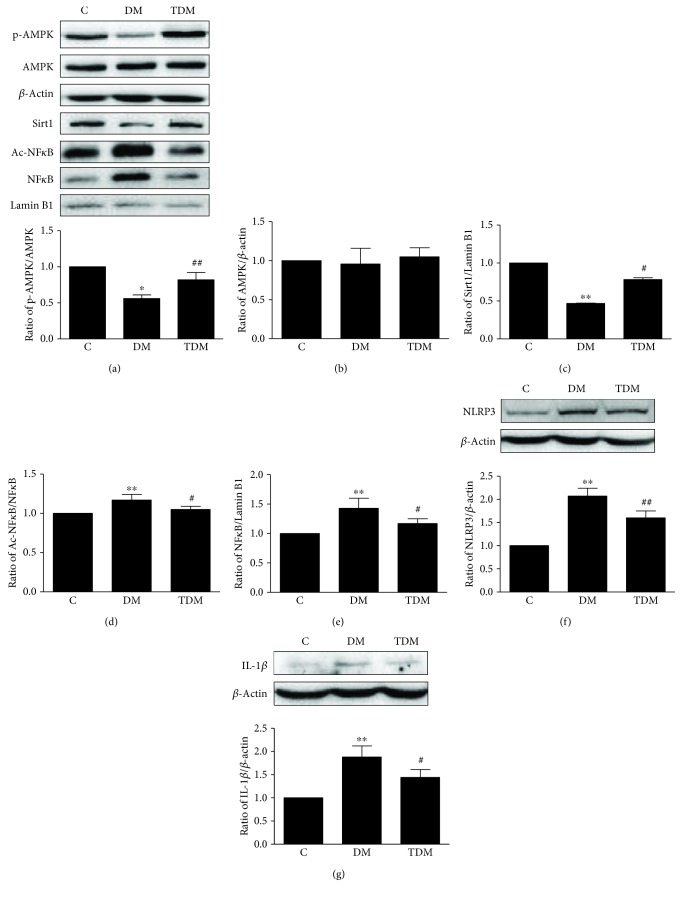
Hippocampal AMPK/Sirt1 and NF*κ*B/NLRP3/IL-1*β* signaling pathways in diabetic rats and the effects of aerobic exercise. Type 2 diabetes significantly decreases the activation of hippocampal AMPK (a) and the level of Sirt1 (c), leading to increased Ac-NF*κ*B (d) and NF*κ*B (e); diabetic rats contain more NLRP3 inflammasomes (f) and IL-1*β* (g). Aerobic exercise intervention significantly increases AMPK activity and Sirt1 concentration; aerobic exercise downregulates the Ac-NF*κ*B and NF*κ*B, leading to decreased levels of the NLRP3 inflammasome and IL-1*β*. ^∗∗^*p* < 0.01, DM group vs. C group; ^#^*p* < 0.05, TDM group vs. DM group.

We investigated whether aerobic exercise can inhibit inflammation in the hippocampus of diabetic rats by analyzing the concentrations and activities of proteins involved in the AMPK/Sirt1 and NF*κ*B/NLRP3/IL-1*β* signaling pathways. The levels of p-AMPK and Sirt1 were lower in DM rats than in C rats and were significantly higher in TDM rats than in DM rats. The levels of Ac-NF*κ*B, NF*κ*B, NLRP3, and IL-1*β* were higher in DM rats than in C rats and were lower in TDM rats than in DM rats (Figure 5). These results indicate that diabetes inhibits the AMPK/Sirt1 pathway and activates the NF*κ*B/NLRP3/IL-1*β* pathway and that aerobic exercise promotes the AMPK/Sirt1 pathway and inhibits inflammation induced by diabetes in the hippocampus.

## 4. Discussion

We found that diabetic rats exhibit memory impairment with decreased levels of synaptic plasticity-associated proteins. Diabetic rats are also marked by deficits in the PI3K/Akt/mTOR and AMPK/Sirt1 signaling pathways and activation of the NF*κ*B/NLRP3/IL-1*β* inflammation pathway in the hippocampus. Aerobic exercise increases the levels of synaptic plasticity-associated proteins, accompanied by activation of the PI3K/Akt/mTOR and AMPK/Sirt1 signaling pathways and by inhibition of the NF*κ*B/NLRP3/IL-1*β* pathway in the hippocampus of diabetic rats. These results indicate that impairment of the insulin signaling pathway, imbalance of energy metabolism, and inflammation may contribute to impaired synaptic plasticity, while aerobic exercise upregulates the expression of synaptic plasticity-associated proteins through regulating these signaling pathways. As these changes of signaling pathways were accompanied by alterations of hippocampal glucose metabolism, we infer that alterations of hippocampal glucose metabolism are involved in the regulation of synaptic plasticity.

### 4.1. Memory Function in Diabetic Rats and the Effects of Aerobic Exercise

We demonstrated memory deficits in diabetic rats with Y maze tests. We found a reduction of the number of actual alternations and the number of maximum alternations compared to control rats in the spontaneous alternation test; additionally, the total number of arm entries and the number of novel arm entries were both lower in diabetic rats than in control rats in the novel object recognition test. These results are consistent with the findings of previous studies. For example, diabetic rats have significantly decreased percent alternation and total arm entries when compared to control rats in the Y maze [20]. Memory deficits of diabetic rats were also observed in the Morris water maze and T maze [21, 22]. These studies confirmed that diabetes is associated with cognitive dysfunction.

Aerobic exercise has been widely reported to recuperate diabetes-associated cognitive dysfunction. For example, de Senna et al. found that 5 weeks of moderate-intensity aerobic exercise improves spatial memory of diabetic rats, shown by longer time spent in exploring the novel object in the novel object recognition test [23]. In our study, aerobic exercise did not significantly change the memory performance of diabetic rats, but the memory performance of diabetic rats showed an improved tendency after 4 weeks of aerobic exercise intervention. We speculate that the difference may be a result of the different aerobic exercise programs or the duration of the exercise training.

### 4.2. Hippocampal Synaptic Plasticity-Associated Proteins in Diabetic Rats and the Effects of Aerobic Exercise

Studies demonstrated that type 2 diabetic animals exhibit deficits in learning and synaptic plasticity, revealed by the decreased spontaneous alternation in the Y maze. Deficits of synaptic plasticity are associated with downregulation of synaptic plasticity-associated proteins. We found that levels of NMDAR1, SYN, and Homer 1 in the hippocampus were lower in diabetic rats than in control rats. Downregulated synaptic plasticity-associated proteins may induce abnormal neurotransmitter release, leading to an imbalance of excitement and inhibition in the central nervous system. Moreover, decreased synaptic plasticity-associated proteins can hinder synaptic vesicle transport and impair information transduction, processing, and storage in the nervous system, eventually leading to abnormal cognitive behavior.

Aerobic exercise is important in promoting the expression of synaptic plasticity-associated proteins. For instance, Maejima et al. reported that 4 weeks of moderate-intensity aerobic exercise increases the expression of BDNF (brain-derived neurotrophic factor), TrkB (tyrosine kinase receptor B), and NMDAR subunit of NR1 in the hippocampus of a senescence-accelerated mouse, but not in an age-matched control mouse [24]. Our previous study showed that 8 weeks of aerobic exercise could elevate the production of hippocampal plasticity-associated proteins such as BDNF and SYN in both healthy and obese rats [18]. Woo et al. also reported that 8 weeks of aerobic exercise upregulated mRNA of BDNF, TrkA, and TrkB, as well as the protein level of BDNF in obese rats [25]. Moreover, a study has reported that running wheel activity increased levels of BDNF in the hippocampus of db/db diabetic mice [26]. We found that aerobic exercise upregulates the expression of synaptic plasticity-associated proteins in the hippocampus of diabetic rats, which was consistent with the previous studies. However, its mechanism is still unclear.

### 4.3. Hippocampal PI3K/Akt/mTOR Signaling Pathway in Diabetic Rats and the Effects of Aerobic Exercise

PI3K and Akt are important molecules of the insulin signaling pathway. In the brain, the PI3K/Akt signaling pathway is closely related to neuronal survival and synaptic plasticity. Activation of Akt promotes the protein translation and regulates the transport of the synaptic plasticity-associated proteins. Studies found that the PI3K/Akt signaling pathway is necessary for BDNF-induced transduction of PSD95 (postsynaptic density protein 95) to dendrites [27]. In addition, PI3K/Akt activation can increase the phosphorylation level of CREB (cAMP-response element-binding protein) and its transcription activity, thus increasing the expression of synaptic plasticity-associated proteins. Inversely, inhibition of the PI3K/Akt signaling pathway reduces the expression of PSD95 [28]. The above studies suggest that the PI3K/Akt signaling pathway is crucial in promoting synaptic plasticity and cognitive function. In fact, studies reported that activation of the PI3K/Akt signaling pathway in the hippocampus of diabetic or AD (Alzheimer's disease) rats can reduce the hyperphosphorylation of tau protein and alleviate cognitive dysfunction [29, 30]. In this study, we found that levels of p-PI3K and p-Akt were significantly lower in the hippocampus of diabetic rats compared to control rats, consistent with the findings of the previous studies, which reported a decreased level of p-InR (insulin receptor) and p-Akt in models with insulin resistance or diabetes [31–34]. The decline of the PI3K/Akt signaling pathway may be a factor of the decreased expression of hippocampal synaptic plasticity-associated proteins in diabetic rats.

Researchers speculated that the impaired hippocampal insulin signaling pathway led to abnormal translation or transportation of synaptic plasticity-associated proteins. However, the cause of the impairment of hippocampal insulin signaling is still unknown. A few studies reported that mitochondrial dysfunction, increased generation of reactive oxygen species, and neuroinflammation may be factors of the impairment of hippocampal insulin signaling [3]. Based on our previous metabolomics research [1], we hypothesize that high glucose in the hippocampus of diabetic rats causes mitochondrial dysfunction, leading to impaired oxidative phosphorylation and overproduction of reactive oxygen species, which can induce oxidative stress and inflammation, contributors to the deficits of the PI3K/Akt signaling pathway.

mTOR is a downstream protein of the PI3K/Akt signaling pathway, also important in synaptic plasticity. Activating mTOR can stimulate mRNA transcription and protein synthesis by phosphorylation of p70s6k and 4EBP2, which can promote the expression of synaptic plasticity-associated proteins, such as PSD95, thus improving memory performance [35–37]; inhibiting mTOR/p70s6k by rapamycin in the CA3 region of the hippocampus impairs long-term memory [38]. In diabetic animal models, the results concerning mTOR activity in the brain are not consistent, possibly caused by different durations or stages of diabetes. In our study, we found that the level of p-mTOR significantly decreased in diabetic rats when compared with control rats, suggesting that mTOR activity is inhibited in diabetic rats. Additionally, the downstream proteins of mTOR, such as p70s6k and 4EBP2, showed lower phosphorylation levels in the hippocampus of diabetic rats compared with control rats. We speculate that PI3K/Akt/mTOR deficits may be a dominant cause of the decline in synaptic plasticity-associated proteins.

Exercise activates the PI3K/Akt/mTOR signaling pathway. Kang and Cho [39] found that 6 weeks of treadmill exercise upregulates the level of p-InR, p-PI3K, and p-Akt in the brains of AD rats. Kim et al. [40] found that 12 weeks of aerobic treadmill exercise also significantly increases the levels of p-PI3K and p-Akt in the hippocampus of diabetic rats. In addition, 6 weeks of exercise increases the number of p-mTOR-positive neurons and astrocytes in the striatum, hippocampus, and amygdala of normal rats compared with the static control group [41]. Fang et al. [42] claimed only 5 days of treadmill exercise can increase the level of p-mTOR in the hippocampus of nonstress rats. Our study found that aerobic exercise significantly increases the levels of p-PI3K and p-Akt in the hippocampus of diabetic rats, as well as the phosphorylation level of p70s6k and 4EBP2. The improvement of the PI3K/Akt/mTOR signaling pathway induced by aerobic exercise may contribute to the increased expression of hippocampal synaptic plasticity-associated proteins in diabetic rats.

Aerobic exercise upregulates the TCA cycle and inhibits the polyol pathway and pentose phosphate pathway in the hippocampus of diabetic rats [1]; these alterations induced by aerobic exercise may reduce the production of reactive oxygen species and oxidative stress, thus promoting the PI3K/Akt signaling pathway. As mTOR is extremely sensitive to metabolic signals such as glucose and amino acids, alterations of hippocampal glucose metabolism induced by aerobic exercise are likely a dominant factor in the activation of mTOR signaling.

### 4.4. Hippocampal AMPK/Sirt1 and NF*κ*B/NLRP3/IL-1*β* Signaling Pathways of Diabetic Rats and the Effects of Aerobic Exercise

AMPK and Sirt1 are vital in maintaining memory and synaptic plasticity. Inhibition of expression or activity of AMPK/Sirt1 through pharmacological or genetic methods can lead to decreased synaptic plasticity and cognitive dysfunction; upregulation of the expression or activity of AMPK/Sirt1 can improve cognitive function. Studies reported that decreased levels of p-AMPK and Sirt1, as well as the decreased expression of BDNF and increased tau phosphorylation and apoptosis in the brain, are simultaneously observed in AD animal models [10–12]. In addition, AMPK activity and Sirt1 expression both show a decrease in diabetic animal models [43, 44]. We observed that the activity of AMPK/Sirt1 in the hippocampus of diabetic rats is lower than that of control rats, consistent with the previous studies. We speculate that decreased AMPK and Sirt1 activity is involved in the development of diabetes-associated cognitive dysfunction and decline in synaptic plasticity-associated proteins.

As important energy-sensing proteins, AMPK and Sirt1 activities are closely related to the cellular energy state. Our previous study confirmed that abnormal glucose metabolism in the hippocampus of diabetic rats induces migration of energy production pathways. Therefore, we think that the activity of AMPK and Sirt1 is affected by alterations in glucose metabolism. In diabetic animals, excessive glucose accumulates in the hippocampus, causing AMPK inhibition to reduce the intermediate metabolites in the TCA cycle and the consumption of electron and oxygen. Therefore, lower AMPK activity in the hippocampus of diabetic rats may be an adaptive response of nerve cells to excess nutrients, aiming to reduce intracellular ROS production.

In addition to their neuroprotective function, AMPK and Sirt1 also participate in controlling inflammation. Sirt1 can deacetylate NF*κ*B (nuclear factor-*κ*B) and decrease its transcription activity. The activated NF*κ*B is translocated to the nucleus and promotes the transcription of inflammatory cytokines [45]. For example, NF*κ*B can promote the expression of the NLRP3 inflammasome, and activation of the NLRP3 inflammasome promotes secretion of the inflammatory cytokine IL-1*β* [46, 47]. IL-1*β* is associated with deficits in hippocampal-dependent memory and synaptic plasticity [18].

Many studies claimed that inflammatory cytokines can cause cognitive dysfunction. Previous research showed that levels of NF*κ*B, TNF-*α*, and IL-1*β* were higher, while protein and mRNA levels of BDNF were lower in the hippocampus and cortex of diabetic rats when compared to control rats. Cognitive dysfunction such as learning and memory impairment was also found in diabetic rats [48]. Our results indicate that diabetes increases the levels of NF*κ*B, NLRP3 inflammasome, and IL-1*β* in the hippocampus, suggesting that activation of diabetes-induced NLRP3 and enhanced secretion of IL-1*β* contribute to the progression of diabetic encephalopathy.

Studies also reported that aerobic exercise affects the activity of AMPK and Sirt1. For example, Kim and Leem found that 3 weeks of aerobic exercise activates AMPK [49]. Steiner et al. found that 8 weeks of exercise upregulates the expression of Sirt1 in the brain [50]. Studies verified the anti-inflammatory effects of aerobic exercise. Cai et al. [18] claimed that 8 weeks of moderate-intensity aerobic exercise promotes the expression of hippocampal synaptic plasticity-associated proteins by alleviating hippocampal endoplasmic reticulum stress and upregulating the Nrf2- (nuclear factor E2-related factor 2-) HO-1 (heme oxygenase-1) signaling pathway, thereby reducing the levels of NLRP3 inflammasomes and IL-1*β* in obese rats; 3 months of treadmill exercise reduces the neuroinflammation of leptin receptor-deficient diabetic mice and reverses diabetes-induced impairments of cognitive function and synaptic plasticity in hippocampal neurons [51].

Our study found that aerobic exercise increases AMPK and Sirt1 and decreases the levels of NF*κ*B, NLRP3 inflammasomes, and IL-1*β* in the hippocampus of diabetic rats. Our results are consistent with the previous findings that NF*κ*B signaling can be regulated by the AMPK and Sirt1 pathway. Therefore, aerobic exercise reduces inflammation and alleviates the synaptic plasticity impairment induced by diabetes. Activation of the AMPK/Sirt1 signaling pathway in the hippocampus of diabetic rats induced by aerobic exercise can enhance the activity of the mitochondrial respiratory chain enzyme and ATPase [52]. This increases the energy supply of neurons and reduces oxidative stress, favorable to the maintenance of neuronal structure and function, enhancing cognitive function. However, we are still not certain about the mechanisms of aerobic exercise leading to the activation of the AMPK/Sirt1 signaling pathway in the hippocampus under diabetes. We hypothesize that alterations of hippocampal glucose metabolism and energy production pathways induced by aerobic exercise are important factors.

## 5. Conclusion

**Figure 6:**
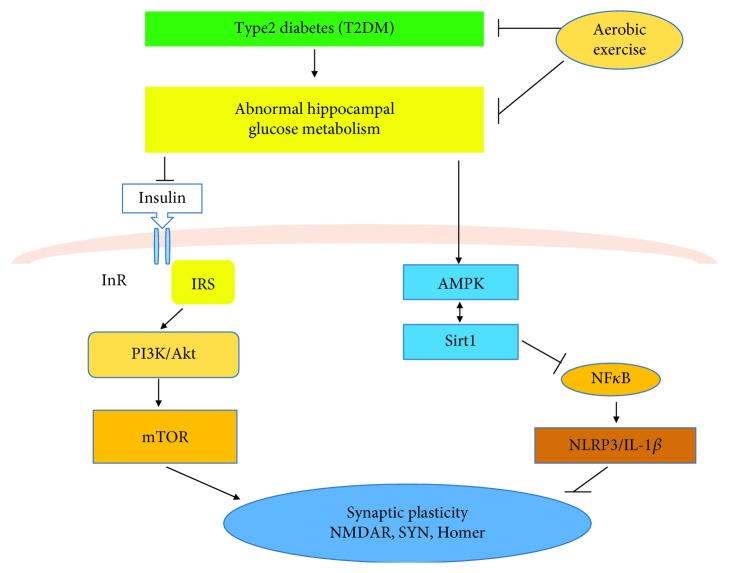
Proposed mechanism by which type 2 diabetes (T2DM) and aerobic exercise can affect the brain and lead to alterations of hippocampal glucose metabolism with subsequent effects on synaptic plasticity-associated proteins. It also suggests a mechanistic pathway by which insulin signaling, energy deficits, and inflammation can regulate hippocampal plasticity and function.

We presented a comprehensive investigation of the protective role of aerobic exercise in a high-fat diet and STZ-induced diabetes and diabetic encephalopathy (Figure 6). We provided evidence that diabetes decreases the expression of synaptic plasticity-associated proteins and accelerates inflammation while aerobic exercise significantly attenuates these effects by upregulating the PI3K/Akt/mTOR pathway and AMPK/Sirt1 pathway or inhibiting the NF*κ*B/NLRP3/IL-1*β* pathway.

## Figures and Tables

**Figure 4:**
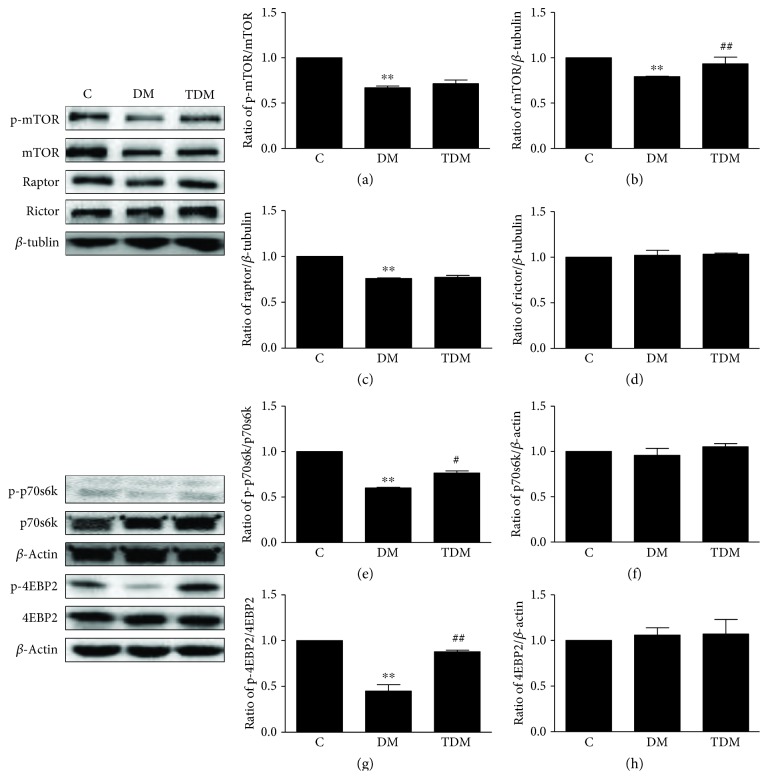
Hippocampal mTOR signaling pathway and its downstream targets in diabetic rats and the effects of aerobic exercise. Type 2 diabetes significantly inhibits the activation of hippocampal mTOR (a) and decreases the levels of mTOR (b) and raptor (c), leading to a decreased activation of p70s6k (e) and 4EBP2 (g). Aerobic exercise does not reverse the reduction in mTOR activity but increases mTOR concentration. Aerobic exercise also promotes p70s6k activity and 4EBP2 activity. ^∗∗^*p* < 0.01, DM group vs. C group; ^#^*p* < 0.05, ^##^*p* < 0.01, TDM group vs. DM group.

**Table 1:** Positive effects of exercise on glucose metabolism in rats.

	C (*n* = 8)	DM (*n* = 9)	TDM (*n* = 8)
FBG (mmol/l)	5.33 ± 0.26	29.73±2.98^∗∗^	21.22 ± 5.18^##^
FINS (*μ*IU/ml)	9.27 ± 0.35	22.81±1.58^∗∗^	17.57 ± 1.55^##^
HOMA-IR	1.85 ± 0.67	29.44±5.10^∗∗^	17.03 ± 3.13^##^

C: control group; DM: diabetes mellitus group; TDM: DM with aerobic exercise training group. Data presented as mean ± SD. FBG: fasting blood glucose; FINS: fasting serum insulin; HOMA-IR: homeostasis model assessment of insulin resistance, calculated by FBG (mmol/l) × FINS (*μ*IU/ml)/22.5. ^∗∗^*p* < 0.01, DM group vs. C group; ^##^*p* < 0.01, TDM group vs. DM group.

**Table 2:** Spontaneous alternation.

	Number of actual alternations	Number of maximum alternations	Index of discrimination
C	5.13 ± 1.25	13.13 ± 2.53	0.46 ± 0.36
DM	2.88±1.36^∗∗^	9.00±2.88^∗∗^	0.42 ± 0.13
TDM	3.75 ± 1.04	9.75 ± 1.98	0.48 ± 0.05

C: control group; DM: diabetes mellitus group; TDM: DM with aerobic exercise training group. Index of discrimination: actual alternation/maximum alternation. Data presented as mean ± SD. ^∗∗^*p* < 0.01, DM group vs. C group.

**Table 3:** Novel object recognition.

	Number of novel arm entries	Total number of arm entries
C	4.00 ± 1.20	9.75 ± 3.01
DM	1.86±0.90^∗∗^	5.71±1.38^∗∗^
TDM	2.13 ± 1.00	7.25 ± 1.67

C: control group; DM: diabetes mellitus group; TDM: DM with aerobic exercise training group. Data presented as mean ± SD. ^∗∗^*p* < 0.01, DM group vs. C group.

## Data Availability

The blood parameters, behavioral indexes, and protein data used to support the findings of this study are included within the article. Previously reported metabolomics data were used to support this study and are available at doi: 10.1016/j.bbr.2017.11.001. These prior studies (and datasets) are cited at relevant places within the text as references.
